# Women from afar: an observational study of demographic characteristics and mortality among foreign‐born women living with HIV in the Women’s Interagency HIV Study (WIHS) in the United States 1994‐2016

**DOI:** 10.1002/jia2.25486

**Published:** 2020-05-21

**Authors:** Adebola Adedimeji, Qiuhu Shi, Lisa Haddad, Susan Holman, Andrew Edmonds, Kathleen Weber, Seble Kassaye, Roksana Karim, Hector Bolivar, Michael Reid, Mirjam‐Colette Kempf, Elizabeth Golub, Donald R. Hoover, Kathryn Anastos

**Affiliations:** ^1^ Department of Epidemiology and Population Health Albert Einstein College of Medicine Bronx NY USA; ^2^ Department of Epidemiology and Community Health School of Health Sciences and Practice New York Medical College Valhalla NY USA; ^3^ Department of Gynecology and Obstetrics Emory University School of Medicine Atlanta GA USA; ^4^ State University of New York Downstate Medical Center Brooklyn NY USA; ^5^ Department of Epidemiology The University of North Carolina at Chapel Hill NC USA; ^6^ Hektoen Institute of Medicine Chicago IL USA; ^7^ Department of Infectious Diseases Georgetown University Washington DC USA; ^8^ Division of Disease Prevention, Policy and Global Health Keck School of Medicine University of Southern California Los Angeles CA USA; ^9^ School of Medicine University of Miami Coral Gables FL USA; ^10^ Institute of Global Health Sciences Division of HIV, Infectious Disease and Global Medicine University of California at San Francisco CA USA; ^11^ Schools of Nursing, Public Health and Medicine University of Alabama at Birmingham Birmingham AL USA; ^12^ Department of Epidemiology Johns Hopkins University Baltimore MD USA; ^13^ Department of Statistics Rutgers University New Jersey NJ USA; ^14^ Department of Medicine Montefiore Medical Center Bronx NY USA

**Keywords:** Immigrants, Foreign‐born Women, HIV, Mortality, WIHS, United States

## Abstract

**Introduction:**

Foreign‐born persons comprise ~13% of the US population. Immigrants, especially women, often face a complex set of social and structural factors that negatively impact health outcomes including greater risk of HIV infection. We described socio‐demographic, clinical and immunological characteristics and AIDs and non‐AIDS death among foreign‐born women living with HIV (FBWLWH) participating in the US Women’s Interagency HIV Study (WIHS) in the US from 1994 to 2016. We hypothesized that FBW will experience higher AIDS‐related mortality compared to US‐born women (USBW).

**Methods:**

The WIHS is a multicenter prospective observational cohort study of mostly women living with HIV (WLWH). The primary exposure in this analysis, which focused on 3626 WLWH, was self‐reported country of birth collapsed into foreign‐born and US born. We assessed the association of birthplace with categorized demographic, clinical and immunological characteristics, and AIDS/non‐AIDS mortality of WLWH, using chi‐squared tests. Proportional hazard models examined the association of birthplace with time from enrolment to AIDS and non‐AIDS death.

**Results:**

Of the 628 FBW, 13% were born in Africa, 29% in the Caribbean and 49% in Latin America. We observed significant differences by HIV status in socio‐demographic, clinical and immunological characteristics and mortality. For both AIDS and non‐AIDS caused deaths FBW WLWH had lower rates of death. Adjusting for year of study enrolment and other demographic/clinical characteristics mitigated FBW’s statistical survival advantage in AIDS deaths Relative Hazard (RH = 0.91 *p* = 0.53), but did not substantively change the survival advantage in non‐AIDS deaths RH = 0.33, *p* < 0.0001).

**Conclusion:**

Foreign‐born WLWH exhibited demographic, clinical and immunological characteristics that are significantly different compared with women born in the US or US territory. After adjusting for these characteristics, the FB WLWH had a significantly lower hazard of non‐AIDS but not AIDS mortality compared to women born in the US or a US territory. These findings of non‐increased mortality can help inform models of care to optimize treatment outcomes among FBWLWH in the United States.

## 1 INTRODUCTION

Over 40 million individuals, about 13% of persons living in United States (US), were born outside of the US [1]. Most originate from countries in South and East Asia, Latin America, sub‐Saharan Africa and the Caribbean and may therefore be considerably different in terms of socio‐cultural characteristics and health status compared with persons born in the US [2]. Studies in the US and Western Europe have suggested that the rate of HIV diagnosis may be higher among foreign‐born persons when compared with native‐born populations [3, 4, 5, 6, 7]. For instance, adult HIV incidence rate in the Caribbean is second only to that in sub‐Saharan Africa [8]. These studies have also highlighted that foreign‐born persons both those who acquired HIV before and after migration have distinct epidemiologic profiles, including higher rates of diagnosis among women, lower rates of injection drug use transmission, higher rates of heterosexual transmission, higher CD4+ T cell counts at diagnosis and lower mortality rates [3, 4].

Foreign‐born populations, especially women are generally disadvantaged in terms of accessing health care due to a complex set of social and structural factors [5, 9, 10, 11], all of which exert powerful negative influences on short‐ and long‐term health outcomes[6, 9, 12, 13, 14, 15, 16, 17]. Studies suggest that foreign‐born women are more susceptible to the less desirable outcomes of HIV due to low socioeconomic and educational status, language barriers, poor access to health providers, lack of transportation, and lack of documented legal status, among others, and may be negatively impacted in accessing or utilizing services because of similar factors [17, 18]. Thus, interventions to meet their needs require an understanding of these multifaceted factors that impact access to care [3, 18].

The situation of foreign‐born WLWH may be particularly challenging in view of this well‐documented complex set of factors that constrains their access to health care and heightens vulnerability to negative long‐term outcomes. Yet, few studies have examined foreign‐born WLWH in the US and how they compare with US‐born WLWH in terms of demographic, clinical and immunologic characteristics and how these factors affect long‐term health outcomes, including mortality. Furthermore, assessing patient‐level outcomes in foreign‐born WLWH in the US can provide critical information regarding AIDS‐related mortality among a particularly vulnerable population who may face challenges in accessing care. We hypothesized that foreign‐born WLWH will experience higher AIDS‐related mortality compared to US‐born women (USBW).

The Women’s Interagency HIV Study (WIHS) is a long‐standing cohort of WLWH and otherwise similarly HIV‐negative women recruited to be representative of the populations of women living with HIV in the US [19, 20, 21]. No study has examined how foreign‐born WIHS participants, who are often grouped into “Black/African‐American, Latina or Asian” despite the heterogeneity and diversity of these population groups, compare with their US born counterparts in terms of long‐term HIV outcomes, especially mortality.

In this study, we thus describe: (i) socio‐demographic, clinical and immunological characteristics; (ii) factors that could impact access to and utilization of health services; specifically access to health insurance and antiretroviral therapy (ART) use; and iii) AIDS and non‐AIDS death of foreign born and native born WIHS WLWH.

## 2 METHODS

### 2.1 Study population

The WIHS is a multicenter observational prevalent cohort study of 3678 WLWH and 1304 HIV‐negative women at risk of HIV infection enrolled in four waves: 1994‐95, 2001‐02, 2011‐12 and 2013‐2015. The WIHS recruited from ten sites: Brooklyn and Bronx New York; Chicago, Illinois; Los Angeles and San Francisco, California; Washington, District of Columbia; Atlanta, Georgia; Birmingham, Alabama; Jackson, Mississippi; Chapel Hill, North Carolina, and Miami, Florida. Women were enrolled based on having a known HIV‐seropositive (prior diagnosis) or HIV‐seronegative status with a history of sexually transmitted infections (STIS) or demographic and behavioural characteristics that increased their susceptibility to HIV acquisition. Details of the study population and recruitment strategies have been previously described [19, 20, 21]. Participants were not deliberately matched on any characteristics prior to enrolment into the study.

### 2.2 Ethical approval and participant interviews

Ethical approval for the study was obtained from the Institutional Review Boards of each of the WIHS principal investigators’ primary institution (see list in acknowledgement). Prior to the interviews, written informed consent was obtained from each participant.

Participants were interviewed and examined semiannually using structured questionnaires administered by trained research staff, and laboratory testing was conducted to assess HIV‐related outcomes [19, 20, 21]. This analysis included all 3626 WLWH who self‐reported on country of birth. All the data used in this analysis were de‐identified.

### 2.3 Exposure and outcome variables

The primary exposure for these analyses was self‐reported country of birth categorized as: US (excluding US territories), US territories or foreign‐born (i.e. birthplace not within the US or a US territory). For this analysis, birthplace was the place the participant was born irrespective of her citizenship or parent’s birthplace. For foreign‐born women, region of birth was categorized as Africa, Caribbean, Latin America or “other” (which was mostly Canada and Europe).

Socio‐demographic covariates examined included self‐reported race/ethnicity, categorized as white (Hispanic and non‐Hispanic together), black (Hispanic and non‐Hispanic together) or other (who mostly self‐identified as Hispanic but not white or black); and other characteristics at enrolment, including age (<30 years, 30 to 40>40), marital status (legal/common law married, never married/ living with partner/other), education (<high school, ≥high school), residence (own house/apartment, parents’ house/living with someone else, rooming/boarding/other), employment (no or yes), annual household income (≤$12,000 or>$12,000), health insurance (no or yes), always see same health provider (no or yes) and year of enrolment into the WIHS (1994 to 1995, 2001 to 2002, 2011 to 2013, 2014+). Clinical variables included in this analysis included: source of HIV acquisition (self‐identified as injection drug use or heterosexual sex/ other) and CD4+ T‐cell count category at enrolment (<200, 200 to 350, 351 to 500, >500 cells/µL), for all of these exposure variables the baseline value taken at study entry was included in models of time to death.

We also included in models of time to death as a time‐dependent variable (being updated at follow‐up visits) HIV viral load (VL) (undetectable or detectable), and type of antiretroviral therapy (ART) currently used (none, monotherapy, combination therapy, or highly active antiretroviral therapy (HAART)). HAART was defined as any three‐drug antiretroviral regimen, one of which must have been a protease inhibitor, a non‐nucleoside reverse transcriptase inhibitor, one of the nucleoside reverse transcriptase inhibitors abacavir or tenofovir, an integrase inhibitor or an entry inhibitor [22]. Mono‐therapy was the use of either one antiretroviral drug; combination therapy included all regimens that did not qualify as HAART or mono‐therapy.

Date of death was ascertained for all participants who died and cause of death was classified as AIDS‐related (including pneumonia/infection), non‐AIDS, or unknown for WLWH using methods that have been previously described, including routine follow‐up by WIHS staff and matching death reports with National Death Index [23, 24].

### 2.4 Statistical analysis

Associations of participants’ birthplace (foreign, US, US‐territory) with categorized baseline characteristics were assessed using the chi‐squared tests [25]. Proportional hazard models [26], which reflect the risk or relative hazard (RH) of dying from a specified cause over a very short period of time, examined the association of birthplace with time from WIHS enrolment to all‐cause mortality. We censored surviving subjects at the last date they were seen in WIHS. However, for the analyses of time to AIDS death, participants who died from non‐AIDS conditions were censored at the date of death and similarly participants whose death was AIDS‐related were censored at the date of death when the outcome was not AIDS death. Successive proportional hazard models assessing the associations of birthplace with time to death were cumulatively adjusted for the following variables (unadjusted analysis is Model 1): *Model 2* = birthplace plus year of WIHS enrolment; *Model 3* = Model 2 plus socio‐demographic characteristics of age, race, education, annual household income, HIV‐risk category and employment status. For WLWH, an additional proportional hazards model was added: *Model 4* = Model 3 plus HIV clinical/treatment variables of CD4 count, health insurance, HIV VL and ART use. Participants enrolled after 2011 were excluded from death analyses because of inadequate follow‐up time.

## 3 RESULTS

Table 1 shows demographic and clinical characteristics of all WLWH (n = 3626) in the WIHS by place of birth.

**Table 1:** Baseline demographic and clinical characteristics of all WLWH in the Women’s Interagency HIV Study 1994 to 2016

	US Born (n = 2899) %	USA Territory (n = 99) %	Foreign Born (n = 628) %	Total (n = 3626) %	*p* value
Age (years)					
<30	464 (16%)	16 (16%)	223 (36%)	703 (19%)	<0.001
30‐40	1250 (43%)	45 (45%)	281 (45%)	1576 (43%)	
>40	1185 (41%)	38 (38%)	122 (19%)	1345 (37%)	
Region of birth			81 (13%)	81 (2%)	
Africa			181 (29%)	181 (5%)	<0.001
Caribbean			310 (49%)	310 (9%)	
Latin America	2899 (100%)			2899 (80%)	
USA		99 (100%)		99 (3%)	
USA Territory			56 (9%)	56 (2%)	
Other			81 (13%)	81 (2%)	
Marital status					
Legally/common law married	584 (20%)	23 (23%)	179 (29%)	786 (22%)	<0.001
Not married/ living with partner/other	1282 (44%)	60 (61%)	264 (42%)	1606 (44%)	
Never married	1024 (35%)	16 (16%)	180 (29%)	1220 (34%)	
Education					
<High School	1003 (35%)	53 (54%)	281 (45%)	1337 (37%)	<0.001
>=High School	1891 (65%)	46 (46%)	346 (55%)	2283 (63%)	
Where are you living now?					
Own house/apartment	1960 (68%)	82 (83%)	447 (71%)	2489 (69%)	<0.001
Parent’s house/with someone else	589 (20%)	11 (11%)	151 (24%)	751 (21%)	
Rooming/boarding/all other	349 (12%)	6 (6%)	29 (5%)	384 (11%)	
Employment status					
No	2203 (76%)	88 (89%)	401 (64%)	2692 (74%)	<0.001
Yes	691 (24%)	11 (11%)	226 (36%)	928 (26%)	
Annual household income					
<$12,000	1719 (61%)	75 (77%)	326 (54%)	2120 (60%)	<0.001
≥$12,000	1099 (39%)	22 (23%)	275 (46%)	1396 (40%)	
Health insurance					
No	410 (14%)	8 (8%)	162 (26%)	580 (16%)	<0.001
Yes	2471 (86%)	91 (92%)	460 (74%)	3022 (84%)	
See same health provider					
No	176 (7%)	6 (6%)	40 (7%)	222 (7%)	0.97
Yes	2437 (93%)	88 (94%)	509 (93%)	3034 (93%)	
Year enrolled in WIHS					
1994 to 1995	1713 (59%)	74 (75%)	263 (42%)	2050 (57%)	<0.001
2001 to 2002	465 (16%)	19 (19%)	253 (40%)	737 (20%)	
2011 to 2014+	721 (25%)	6 (6%)	112 (18%)	839 (23%)	
HIV risk category*					
Intravenous drug use	795 (28%)	25 (26%)	19 (3%)	839 (23%)	<0.001
Heterosexual/other	2091 (72%)	73 (74%)	602 (97%)	2766 (77%)	
CD4 category*					
<200, cells/µL	605 (22%)	31 (33%)	106 (17%)	742 (21%)	0.062
200 to 350 cells/µL	579 (21%)	19 (20%)	132 (21%)	730 (21%)	
351 to 500 cells/µL	583 (21%)	21 (22%)	138 (22%)	742 (21%)	
>500 cells/µL	1043 (37%)	24 (25%)	245 (39%)	1312 (37%)	
Viral load*					
Undetectable	600 (21%)	17 (18%)	176 (28%)	793 (22%)	0.0012
Detectable	2211 (79%)	80 (82%)	444 (72%)	2735 (78%)	
ART use at enrolment*					
None	1030 (36%)	22 (22%)	193 (31%)	1245 (34%)	<0.001
Mono‐therapy	565 (19%)	26 (26%)	91 (15%)	682 (19%)	
Combination therapy	504 (17%)	32 (32%)	98 (16%)	634 (17%)	
HAART	799 (28%)	19 (19%)	245 (39%)	1063 (29%)	
Death					
Proportion Alive, %	1886 (65%)	57 (58%)	544 (87%)	2487 (69%)	<0.001
Cause of death (n = 1139), %					
Unknown	82 (8%)	5 (12%)	3 (4%)	90 (8%)	<0.0049
AIDS	396 (39%)	20 (48%)	54 (64%)	470 (41%)	
Non‐AIDS	392 (39%)	12 (29%)	17 (20%)	421 (37%)	
Pneumonia or infection	143 (14%)	5 (12%)	10 (12%)	158 (14%)	

* Asterisk indicates significant levels.

Most women at study entry were between 30 and 40 years old, unmarried, but living with a partner. The majority had a high school education, lived in their own home or apartment, and had health insurance, which may have contributed to the high proportion reporting seeing the same health provider each time they seek care. At enrolment, few women were employed and most had household incomes of ≤$12,000 annually. Of the 628 FBW, the largest proportion was born in Latin America, followed by the Caribbean, Africa and other regions. At enrolment, heterosexual intercourse was the most likely source of HIV acquisition reported by most women followed by women who acquired HIV likely through injection drug use. Slightly higher than one‐third (37%) of WLWH had CD4 count> 500 cells/µL; viral load was detectable in more than two‐thirds (78%) and 34% of participants were not using any antiretroviral therapy at enrolment. More than half of all deaths was due to a non‐AIDS related cause, including pneumonia compared with 41% who died of an AIDS‐related cause.

Table 2 shows demographic and clinical characteristics of foreign‐born WLWH. About 38% were not using any antiretroviral therapy at enrolment into the study. Marital status, employment, income, health insurance, level of education, year of enrolment in WIHS and HIV‐risk group all differed significantly by region (*p*=<0.001). Most foreign‐born WLWH had a detectable viral load regardless of the region of birth and those with CD4 count <=350/µL ranges from 29% among those born in Africa to 38% of those born in the Caribbean, 42% of those born in Latin America and 32% of those born in “Other” regions (*p* = 0.011). Heterosexual intercourse was the only HIV risk category for nearly all FB WLWH (*p*=<0.001).

**Table 2:** Baseline demographic and clinical characteristics of all foreign‐born WLWH in the Women’s Interagency HIV Study 1994‐2016

	Africa (n = 81) %	Caribbean (n = 181) %	Latin America (n = 310) %	Other (n = 56) %	*p* value
ART use at enrolment among HIV positive					
None	28 (35%)	65 (36%)	79 (25%)	21 (38%)	0.005
Mono	6 (7%)	27 (15%)	50 (16%)	8 (15%)	
Combo	4 (5%)	26 (14%)	58 (19%)	10 (18%)	
HAART	43 (53%)	63 (35%)	123 (40%)	16 (29%)	
Marital status					
Legally/common law married	35 (44%)	35 (19%)	95 (31%)	14 (25%)	<0.001
Not married/ living with partner/other	16 (20%)	66 (37%)	158 (51%)	24 (44%)	
Never married	29 (36%)	79 (44%)	55 (18%)	17 (31%)	
Where are you living now?					
Own house/apartment	58 (72%)	135 (75%)	213 (69%)	41 (75%)	0.794
Parents house/with someone else	20 (25%)	40 (22%)	80 (26%)	11 (20%)	
Rooming/boarding/all other	3 (4%)	6 (3%)	17 (5%)	3 (5%)	
Employment status					
No	37 (46%)	112 (62%)	222 (72%)	30 (55%)	<0.001
Yes	44 (54%)	69 (38%)	88 (28%)	25 (45%)	
Income					
<$12,000	34 (46%)	82 (46%)	194 (65%)	16 (30%)	<0.001
>=$12,000	40 (54%)	95 (54%)	103 (35%)	37 (70%)	
See same health provider					
No	10 (16%)	15 (9%)	14 (5%)	1 (2%)	0.007
Yes	52 (84%)	147 (91%)	262 (95%)	48 (98%)	
Health Insurance					
No	29 (37%)	22 (12%)	98 (32%)	13 (25%)	<0.001
Yes	50 (63%)	159 (88%)	211 (68%)	40 (75%)	
Education					
<High School	8 (10%)	64 (35%)	200 (65%)	9 (16%)	<0.001
>=High School	73 (90%)	117 (65%)	109 (35%)	47 (84%)	
Age, years					
<30	31 (38%)	60 (33%)	119 (39%)	13 (23%)	0.197
30 to 40	38 (47%)	83 (46%)	134 (43%)	26 (46%)	
>40	12 (15%)	37 (21%)	56 (18%)	17 (30%)	
Year enrolled in WIHS					
1994 to 1995	17 (21%)	72 (40%)	142 (46%)	32 (57%)	<0.001
2001 to 2002	40 (49%)	70 (39%)	130 (42%)	13 (23%)	
2011 to 2014+	24 (30%)	39 (22%)	38 (12%)	11 (20%)	
Viral load					
Undetectable	31 (39%)	48 (27%)	87 (28%)	10 (18%)	0.065
Detectable	49 (61%)	130 (73%)	220 (72%)	45 (82%)	
CD4 category					
<200 cells/µL	2 (3%)	35 (20%)	60 (19%)	9 (17%)	0.011
200 to 350 cells/µL	21 (26%)	32 (18%)	71 (23%)	8 (15%)	
351 to 500 cells/µL	23 (29%)	34 (19%)	71 (23%)	10 (19%)	
>500 cells/µL	34 (43%)	76 (43%)	108 (35%)	27 (50%)	
HIV risk group					
Intravenous drug use		3 (2%)	6 (2%)	10 (18%)	<0.001
Heterosexual/other	79 (100%)	177 (98%)	301 (98%)	45 (82%)	
Death					
Proportion Alive, %	78 (96%)	150 (83%)	265 (85%)	51 (91%)	0.018
Cause of death, %					
Unknown		1 (3%)	2 (4%)		0.474
AIDS		19 (61%)	31 (69%)	4 (80%)	
Non‐AIDS	2 (67%)	6 (19%)	8 (18%)	1 (20%)	
Pneumonia or infection	1 (33%)	5 (16%)	4 (9%)		

### 3.1 Mortality

In unadjusted models (Tables 3 and 4; Model 1), FBW had a lower hazard of death during follow‐up. The HR was 0.50 (95% CI: 0.39, 0.65), *p* < 0.001 for AIDS death and 0.20 (95% CI: 0.12 to 0.32), *p* < 0.001 for non‐AIDS death. Thus, for example FBW had an estimated only half (0.50) the risk of dying from AIDS and one‐fifth (0.20) from non‐AIDS causes versus US born women. Adjusting for enrolment date (Model 2, Table 3) mitigated the FBW’s survival advantage in AIDS‐related deaths: HR: 0.68 (95% CI: 0.52 to 0.88), *p* = 0.0036, compared to USBW. In the fully adjusted model incorporating clinical as well as socio‐demographic factors (Model 4, Table 3), the HR was further attenuated and not statistically significant: HR: 0.91, (95% CI: 0.67 to 1.23), *p* = 0.53. Other factors significantly associated with a lower hazard of AIDS death included later enrolment in WIHS (HR: 0.46; 95% CI: 0.33 to 0.65; *p* < 0.001), and being employed (HR: 0.51; 95% CI 0.39 to 0.67; *p*=<0.001). As time‐dependent variables, having detectable (vs. undetectable) VL (HR 3.79, 95% CI 2,78 to 5.18, *p = 0*.0001) and CD4+ T‐cell counts less than 500 cells/µL (vs. above 500 cells/µL) were associated with higher hazard of death. Those with CD4+ T‐cell count < 200 cells/µL: HR: 7.74 (vs. above 500 cells/µL); 95% CI: 5.91 to 10.13; *p* < 0.001) had the highest hazard of AIDS‐related death.

**Table 3:** Predictors of AIDS death in WLWH in the Women’s Interagency HIV Study 1994 to 2016

Model	Variable	HR	95%CI	*p*‐value
Model 1	US Territory versus US	1.15	(0.77, 1.71)	0.49
	Foreign versus US	0.50	(0.39, 0.65)	<0.0001
Model 2	US Territory versus US	1.11	(0.74, 1.66)	0.61
	Foreign versus US	0.68	(0.52, 0.88)	0.0036
	Enrollment:01‐02 versus ‐94‐95^a^	0.23	(0.17, 0.31)	<0.0001
Model 3	US Territory versus US	1.18	(0.76, 1.83)	0.47
	Foreign versus US	0.87	(0.64, 1.17)	0.35
	Age 30 to 40 versus <30	1.46	(1.15, 1.86)	0.002
	Age> 40 vs. <30	1.57	(1.21, 2.04)	0.0007
	Race: White versus black	0.85	(0.68, 1.06)	0.16
	Race: Other versus black	0.78	(0.61, 0.99)	0.048
	Education: < HS versus >= HS	1.09	(0.91, 1.29)	0.35
	Enrollment:01‐02 versus‐94‐95^a^	0.22	(0.16, 0.31)	<0.0001
	Risk CAT: IDU versus Other	0.91	(0.76, 1.09)	0.31
	Employ: Yes versus No	0.42	(0.32, 0.55)	<0.0001
	Income: <12K versus >= 12K	1.00	(0.82, 1.20)	0.96
	Insurance: Yes versus No	1.16	(0.92, 1.46)	0.21
Model 4	US Territory versus US	1.02	(0.66, 1.59)	0.93
	Foreign VS US	0.91	(0.67, 1.23)	0.53
	Age 30 to 40 versus <30	1.29	(1.01, 1.65)	0.04
	Age> 40 versus <30	1.29	(0.98, 1.68)	0.065
	CD4 < 200 versus >500	7.74	(5.91, 10.13)	<0.0001
	CD4 200 to 349 versus >500	1.91	(1.40, 2.59)	<0.0001
	CD4 350 to 499 versus >500	1.68	(1.23, 2.31)	0.0013
	Race: White versus black	0.87	(0.70, 1.10)	0.25
	Race: Other versus black	0.87	(0.68, 1.12)	0.29
	Education: < HS versus >= HS	1.15	(0.97, 1.37)	0.11
	Enrollment:01‐02 versus‐94‐95^a^	0.46	(0.33, 0.65)	<0.0001
	Risk CAT: IDU versus other	1.02	(0.85, 1.22)	0.86
	Employ: Yes versus no	0.51	(0.39, 0.67)	<0.0001
	Income: <12K versus >= 12K	1.02	(0.84, 1.24)	0.83
	Insurance: Yes versus no	1.10	(0.87, 1.39)	0.41
	VL: Detectable versus undetectable^b^	3.79	(2.78, 5.18)	<0.0001
	ART: Mono versus none^b^	1.89	(1.43, 2.46)	<0.0001
	ART: Combo versus none^b^	1.30	(1.01, 1.69)	0.044
	ART: HAART versus none^b^	0.65	(0.51, 0.82)	0.0003

^a^ Women enrolled after 2011 were excluded because of inadequate follow up time.

^b^ As a time dependent variable.

**Table 4:** Predictors of Non‐AIDS Death in WLWH in the Women’s Interagency HIV Study 1994 to 2016

Model	Variable	HR	95%C.I.	*p*‐value
Model 1	US Territory versus US	0.77	(0.43, 1.37)	0.3700
	Foreign versus US	0.20	(0.12, 0.32)	<0.0001
Model 2	US Territory versus US	0.76	(0.43, 1.34)	0.34
	Foreign versus US	0.22	(0.14, 0.36)	<0.0001
	Enrolment: 01‐02 versus‐94‐95^a^	0.59	(0.44, 0.79)	0.0004
Model 3	US Territory versus US	0.59	(0.31, 1.12)	0.10
	Foreign versus US	0.30	(0.17, 0.53)	<0.0001
	Age 30 to 40 versus <30	1.160	(0.83, 1.61)	0.39
	Age> 40 versus <30	2.34	(1.68, 3.26)	<0.0001
	Race: White versus black	1.12	(0.86, 1.45)	0.41
	Race: Other versus black	1.00	(0.74, 1.35)	0.99
	Education: < HS versus >= HS	1.42	(1.16, 1.75)	0.0008
	Enrollment:01‐02 versus‐94‐95^a^	0.79	(0.58, 1.07)	0.13
	Risk CAT: IDU versus other	1.93	(1.56, 2.39)	<0.0001
	Employ: Yes versus no	0.45	(0.32, 0.64)	<0.0001
	Income: <12K versus >= 12K	1.34	(1.04, 1.72)	0.025
	Insurance: Yes versus no	1.27	(0.94, 1.69)	0.12
Model 4	US Territory versus US	0.61	(0.32, 1.16)	0.13
	Foreign versus US	0.33	(0.19, 0.57)	<0.0001
	Age 30 to 40 versus <30	1.19	(0.85, 1.67)	0.30
	Age> 40 versus <30	2.37	(1.70, 3.20)	<0.0001
	CD4 < 200 versus >500	1.48	(1.10, 1.99)	0.01
	CD4 200‐349 versus >500	1.49	(1.14, 1.94)	0.0034
	CD4 350‐499 versus >500	1.00	(0.75, 1.34)	0.99
	Race: White versus black	1.15	(0.89, 1.50)	0.28
	Race: Other versus black	1.00	(0.74, 1.36)	0.99
	Education: < HS versus >= HS	1.43	(1.17, 1.76)	0.0006
	Enrolment:01‐02 versus‐94‐95^a^	0.98	(0.71, 1.34)	0.88
	Risk CAT: IDU versus other	1.97	(1.59, 2.44)	<0.0001
	Employ: Yes versus no	0.48	(0.34, 0.68)	<0.0001
	Income: <12K versus >= 12K	1.31	(1.02, 1.69)	0.037
	Insurance: Yes versus no	1.30	(0.97, 1.74)	0.085
	VL: Detectable versus undetectable^b^	1.71	(1.33, 2.20)	<0.0001
	ART: Mono versus none^b^	0.97	(0.58, 1.63)	0.92
	ART: Combo versus none^b^	1.19	(0.82, 1.71)	0.37
	ART: HAART versus none^b^	0.79	(0.61, 1.03)	0.083

^a^ Women enrolled after 2011 were excluded because of inadequate follow‐up time.

^b^ As a time‐dependent variable.

In analyses of non‐AIDS deaths, adjusting for enrolment year (Model 2, Table 4) did not substantively change the survival advantage of being FBW compared with USBW from (HR: 0.59; 95% CI: 0.44 to 0.79; *p* < 0.004), and in the model fully adjusted for socio‐demographic and clinical parameters (Model 4, Table 4) the protective effect of being foreign‐born was little changed (HR: 0.33; 95% CI: 0.19 to 0.57; *p* < 0.001)**.** Other predictors of non‐AIDS death in the fully adjusted model included being employed**,** age> 40 years old, history of injection drug use and CD4 count above 500 cells/μL.

## 4 DISCUSSION

In this study of the demographic characteristics and mortality of foreign‐born WLWH in the WIHS, we found that most were young and educated, but low income and often unemployed. Age, education, income, employment, social support and access to health services as well as changing HIV treatment guidelines have been highlighted as critical determinants of health and some studies have suggested that these micro and macro factors exert powerful influences on long‐term outcomes and death among foreign born persons[14, 15, 16, 17, 27, 28, 29]. Our findings reveal that these demographic characteristics, including access to health insurance and use of ART are all significantly associated with birthplace and are critical to long‐term health outcomes. As other studies [30, 31] have reported, access to health insurance may be particularly important for immigrants in general and foreign‐born WLWH in particular to enable them receive continuous health care that can contributing to continuity in primary care to improved long‐term outcomes. Although the majority of WIHS foreign‐born participants having health insurance, and nearly all had access to continuity in primary care, this high level of access may not be generalizable to all foreign‐born persons living with HIV in the US.

The clinical characteristics of foreign‐born WLWH in the WIHS are similar to those previously reported in the literature [32, 33]. HIV transmission in FBW was almost exclusively through heterosexual/other activity in contrast to women born in the US among whom more than a quarter reported injection drug use. Nearly one‐third of foreign born WLWH were not using ART when they enrolled in WIHS, although some of these women were enrolled in 1994, prior to the availability of HAART. Compared to women born in the US or a US territory, foreign‐born WLWH were more likely to have higher CD4+ T cell counts, undetectable VLs at enrolment and a lower hazard of death, in part reflecting their later enrolment when HAART became widely available.

Contrary to our hypothesis, we found that FBW did not have higher death rates compared with women born in the US. In fact, being foreign‐born substantially protected against non‐AIDS death, even in adjusted models. Epidemiological and social science research [34, 35] has consistently documented the “healthy immigrant effect” in which foreign born persons are shown to have better outcomes across a range of health conditions compared to native‐born populations, although some authors have argued that selection bias may account for this observed salutary effect among immigrants [30, 36, 37]. Our findings on hazards of AIDS and non‐AIDS death in foreign‐born WLWH, support the conclusion of studies of the healthy immigrant effect; that being foreign‐born may confer a survival advantage.

The findings presented here are limited by a number of factors, which warrant caution in the interpretation of the results. First, the manner in which WIHS participants were recruited, without a consideration for a place of birth may have introduced selection bias. Thus, foreign born WLWH represented in the WHIS may not accurately reflect the general population of foreign‐born persons living with HIV in the US. Second, WLWH were previously diagnosed with HIV before they were recruited into the study, hence we do not have information on testing rates or how these results will differ if HIV status was unknown. Third, we did not assess how length of HIV diagnosis and access to clinic and non‐clinic‐based support services impacted on the outcomes. Perhaps, addressing these could have impacted the findings presented here. Similarly, we did not undertake any analysis to consider the effect of country of origin or how changing rates of HIV infection and access to treatment or AIDS mortality may have changed among WLWH over time. These are all issues that warrant further investigation. Nevertheless, foreign born WLWH represented in the WIHS cohort demonstrate significant demographic and clinical outcomes when compared to women born in the US or a US territory.

## 5 CONCLUSION

Compared to women born in the US or US territory, foreign born WLWH are demographically, clinically and immunologically different. Injecting drug use was rarely a route of HIV acquisition for foreign‐born women. Contrary to our hypothesis, foreign‐born WLWH have a significantly lower hazard of non‐AIDS death compared with US‐born WLWH. The different demographic, clinical and immunological characteristics exhibited by foreign‐born WIHS participants can have important public health implications for their care and treatment and help inform models of care to optimize treatment outcomes of foreign‐born WLWH.

## COMPETING INTERESTS

The authors have no competing interest to declare.

## AUTHORS’ CONTRIBUTIONS

AA wrote initial drafts and revised the manuscript. AA., QS., DH. and KA. performed research, curated data, performed analysis, and contributed to initial drafts, revisions. LH., SH., AE., KW., SK., RK., HB., MR., MCK. and EG contributed to initial drafts and read revised drafts of the manuscript. All authors have read and approved the manuscript.

## ACKNOWLEDEGMENTS

Data in this manuscript were collected by the Women’s Interagency HIV Study (WIHS). The contents of this publication are solely the responsibility of the authors and do not represent the official views of the National Institutes of Health (NIH). WIHS (Principal Investigators): UAB‐MS WIHS (Mirjam‐Colette Kempf and Deborah Konkle‐Parker), U01‐AI‐103401; Atlanta WIHS (Ighovwerha Ofotokun, Anandi Sheth and Gina Wingood), U01‐AI‐103408; Bronx WIHS (Kathryn Anastos and Anjali Sharma), U01‐AI‐035004; Brooklyn WIHS (Deborah Gustafson and Tracey Wilson), U01‐AI‐031834; Chicago WIHS (Mardge Cohen and Audrey French), U01‐AI‐034993; Metropolitan Washington WIHS (Seble Kassaye and Daniel Merenstein), U01‐AI‐034994; Miami WIHS (Maria Alcaide, Margaret Fischl and Deborah Jones), U01‐AI‐103397; UNC WIHS (Adaora Adimora), U01‐AI‐103390; Connie Wofsy Women’s HIV Study, Northern California (Ruth Greenblatt, Bradley Aouizerat, and Phyllis Tien), U01‐AI‐034989; WIHS Data Management and Analysis Center (Stephen Gange and Elizabeth Golub), U01‐AI‐042590; Southern California WIHS (Joel Milam), U01‐HD‐032632 (WIHS I – WIHS IV). The WIHS is funded primarily by the National Institute of Allergy and Infectious Diseases (NIAID) with additional co‐funding from the Eunice Kennedy Shriver National Institute of Child Health and Human Development (NICHD), the National Cancer Institute (NCI), the National Institute on Drug Abuse (NIDA), and the National Institute on Mental Health (NIMH). Targeted supplemental funding for specific projects is also provided by the National Institute of Dental and Craniofacial Research (NIDCR), the National Institute on Alcohol Abuse and Alcoholism (NIAAA), the National Institute on Deafness and other Communication Disorders (NIDCD) and the NIH Office of Research on Women’s Health. WIHS data collection is also supported by UL1‐TR000004 (UCSF CTSA), UL1‐TR000454 (Atlanta CTSA), and P30‐AI‐050410 (UNC CFAR).
